# Mixed-Methods Investigation of Rural Emergency Medical Services ST-Elevation Myocardial Infarction Time to Percutaneous Coronary Intervention: High- vs Low-Performing Agencies

**DOI:** 10.5811/westjem.43536

**Published:** 2025-07-18

**Authors:** Michael Supples, McKenna E. Gallagher, Nicklaus P. Ashburn, Anna C. Snavely, Ashley E. Strahley, Chadwick D. Miller, Simon A. Mahler, Jason P. Stopyra

**Affiliations:** * Wake Forest University School of Medicine, Department of Emergency Medicine, Winston-Salem, North Carolina; † Wake Forest University School of Medicine, Department of Biostatistics and Data Science, Winston-Salem, North Carolina; ‡ Wake Forest University School of Medicine, Department of Social Sciences and Health Policy, Winston-Salem, North Carolina; § Wake Forest University School of Medicine, Department of Epidemiology and Prevention, Winston-Salem, North Carolina; #,|| Wake Forest University School of Medicine, Department of Implementation Science, Winston-Salem, North Carolina

## Abstract

**Background:**

Patients with ST-elevation myocardial infarction (STEMI) cared for by rural emergency medical services (EMS) agencies commonly do not have first medical contact-to-percutaneous coronary intervention (PCI) time within the recommended goal of 90 minutes. In this study we identify factors associated with performance variation among rural EMS agencies in first medical contact-to-PCI time.

**Methods:**

In this explanatory, sequential, mixed-methods study, we ranked eight rural county EMS agencies by continuous first medical contact-to-PCI time, accounting for loaded mileage, using data from a regional STEMI registry (2016–2019). A qualitative researcher conducted 28, one-hour, semi-structured interviews from January– March 2021 with the EMS director, training officer, medical director, and four paramedics at the top two high- and bottom two low-performing rural EMS agencies. Key informants were blinded to agency STEMI performance. Interviews were structured to identify positive deviance by exploring agencies’ clinical approach to patients with chest pain, their organizational culture, structure, and quality improvement (QI) activities regarding STEMI care, and recommendations for improving STEMI performance. Interviews were digitally recorded and transcribed verbatim by a professional transcription service. We established a codebook and performed a thematic analysis using an inductive approach. We summarized and compared data across agencies to identify commonalities and differences between high- and low-performing agencies. Findings were reviewed and validated by an expert panel.

**Results:**

The top two highest-performing EMS agencies had a median first medical contact-to-PCI time of 79 minutes (interquartile range [IQR] 65–91) minutes vs 98 minutes (IQR 82–120) among the bottom two lowest-performing agencies, *P*<.001. Both high- and low-performing agencies identified issues with electrocardiogram (ECG) transmitting technology and cumbersome hospital activation communications. However, top-performing agencies shared a culture that encourages early EMS activation of the cardiac catheterization lab after STEMI recognition. Top-performing agencies also placed a higher value on QI and training. These agencies prioritized mission and chain of command over staff relationships/interpersonal bonds; have stable, strong leadership; provide opportunities for career advancement; and collaborate with community leaders.

**Conclusion:**

Top-performing rural EMS agencies for STEMI care promote early activation, have a strong chain of command, are mission focused, and have a greater focus on quality improvement and training.

## INTRODUCTION

ST-elevation myocardial infarction (STEMI) is a life-threatening condition in which rapid treatment is the key determinant of improved outcomes.^1^,^2^–^4^ The time between first medical contact and percutaneous coronary intervention (PCI) is linearly associated with mortality.^5^–^8^ The American College of Cardiology/American Heart Association (ACC/AHA) guidelines recommend a first medical contact-to-device time of ≤90 minutes.^2^ Rural emergency medical services (EMS) agencies have variable success in achieving the recommended 90-minute PCI time goal. Rural Americans are less likely to receive timely PCI than urban Americans.^9^–^13^ The disparity in timely PCI drives excess morbidity and mortality among rural patients with STEMI.^13^,^14^ The US Centers for Disease Control and Prevention, Department of Health and Human Services Healthy People 2020, and the Centers for Medicare & Medicaid Services have identified health equity and improving cardiovascular health for rural Americans as urgent goals.^15^,^16^ Further, a goal of EMS Agenda 2050 is the delivery of equitable care regardless of rurality.^17^ Strategies to leverage rural EMS systems to reduce total ischemic time are needed.^18^–^22^

Achieving PCI time goals is multifactorial, including emergency medical services (EMS) agency structure and organizational culture among broader, systems-based factors such as in-hospital process performance and culture.^23^ Organizational culture—defined as a set of shared values, beliefs, and assumptions within an organization that influences how people within that organization behave—has been associated with both cardiovascular mortality and disease-specific outcomes.^24^–^29^ The performance of an EMS system is a key determinant in first medical contact to PCI and improving patient outcomes; however, the impact of agency structure and organizational culture related to STEMI care is unclear.^9^,^10^,^30^–^37^

To address this key evidence gap, we sought to identify the key organizational and behavioral attributes associated with high performance. In this study we quantitatively identified high and low performers based on first medical contact-to-PCI time and then investigated practices that drive differences in performance using qualitative methods.^38^ We hypothesized that organizational culture (eg, teamwork, communication), structure (eg, leadership, training), and practices (eg, meeting benchmarks, interventions) for care of patients with STEMI impact the timeliness of PCI.

## METHODS

### Study Design

We conducted an explanatory, sequential, mixed-methods study of rural STEMI patients with previously published methods.^39^ We used regional STEMI registry data (2016–2019) to quantitatively identify the top two- and bottom two- performing EMS agencies based on continuous first medical contact-to-PCI time. Key informant interviews were conducted at these EMS agencies to investigate organization culture, structure, and practices under the positive deviance framework. Positive deviance is a behavioral research approach based on the concept that groups of organizations performing a similar task have certain members who are high performers relative to their peers, based on specific attributes that allow them to perform more effectively despite having similar resources as other group members.^40^ This study was approved by the Wake Forest University Institutional Review Board. We obtained waivers of informed consent for individual patients included in the registry. This study was registered at ClinicalTrials.gov (NCT04381260).

**Population Health Research Capsule**

What do we already know about this issue?

*Rural patients with ST-elevation myocardial infarction (STEMI) under emergency medical services (EMS) care often do not achieve timely reperfusion, exposing them to increased morbidity and mortality*.

What was the research question?

*What organizational and behavioral factors distinguish high- vs low-performing rural EMS agencies in STEMI timeliness?*

What was the major finding of the study?

*High vs low-performing EMS agencies had median first medical contact to percutaneous coronary intervention time of 79 vs. 98 minutes (−20 min, 95% CI −31 to −10, P<.001)*.

How does this improve population health?

*Findings highlight actionable EMS practices to improve STEMI care in rural settings*.

### Study Setting

Patients were accrued from eight rural EMS agencies in the Piedmont region of North Carolina transporting patients to one of three PCI centers. Agencies continually operated at the paramedic level and received medical direction from a board-certified emergency physician. Ambulance crew configuration was variable but always included at least one paramedic. All agencies had the authority to activate the catheterization (cath) lab, but this activation could be canceled by either the emergency physician or the cardiologist upon review of the transmitted electrocardiogram (ECG). Of note, all EMS agencies had the ability to transmit ECGs. The EMS agencies were selected for participation based on their location in a rural county as defined by the 2014 US Census Bureau American Community Survey.^41^ Descriptive information (number of annual patient transports, staffing and staffed ambulances in a 24-hour period) of the eight rural county EMS agencies^23^ is provided in Supplemental Table 1. The PCI centers were Atrium Health Wake Forest Baptist in Winston-Salem, NC, with 821 licensed beds and full specialty/subspecialty availability; High Point Medical Center in High Point, NC, with 351 licensed beds and extensive specialty/subspecialty availability; and Moses H. Cone Memorial Hospital in Greensboro, NC, with 517 licensed beds and extensive specialty/subspecialty availability. The EMS agencies transport patients with STEMI to the nearest PCI center. The STEMI protocol used by the EMS agencies is provided in Supplemental Figure 1.

### Population

We included patients who were ≥ 18 years, transported to one of the three participating PCI centers by one of eight rural-county EMS agencies, and received primary PCI for STEMI. We excluded interfacility transports and patients with prehospital cardiac arrest.

#### Agency Performance

The 2016–2019 regional STEMI data registry defined EMS first medical contact-to-PCI time as patient initial contact time (recorded by EMS personnel after arrival on scene) to first device activation time (recorded in the National Cardiovascular Data Registry) in minutes. First medical contact-to-PCI time was the key quantitative outcome. Several secondary process outcomes were also defined: dispatch time, the period from when the 9-1-1 call was received to when EMS was dispatched; response time, the period from when EMS was dispatched to arrival on scene; scene time, the period from EMS arrival on scene to EMS departure from scene; ECG time, the period from EMS arrival to first 12-lead ECG being performed; activation time, the period from first 12-lead ECG to when the PCI center team was activated (PCI activation based on EMS pre-activation at the discretion of the destination hospital); transport time, the period from EMS departure from scene to arrival at destination. Total EMS time was the time from EMS arrival on scene to arrival at the hospital, while door-to-balloon time was defined as the time of EMS arrival at destination to PCI time. Loaded mileage was defined as the distance from the incident scene to hospital destination.

### Quantitative Analysis

Among the eight included rural EMS agencies, we ranked their performance on a scale from 1–8 as measured by continuous first medical contact-to-PCI time. Robust regression was used to generate the rankings while adjusting for loaded mileage. We used this technique instead of ordinary least squares regression as it is less sensitive to outliers; thus, no outliers were removed from the analysis.^42^ The two top- and bottom-performing agencies were identified to participate in the qualitative portion of this study. All agencies were staffed by paid EMS professionals, without volunteer members. (Additional first-responder care was provided by local fire and rescue teams that are supplemented with volunteers.) We summarized time metrics using median and interquartile range (IQR) and used Wilcoxon rank-sum tests to compare between the top- and bottom-performing agencies. Hodges–Lehmann estimators were also calculated for the shift in location of the distribution of times for patients in the highest two ranked agencies compared to the lowest two ranked agencies. We used Kruskal-Wallis tests to compare time metrics across all four agencies.

#### Qualitative Data Collection

Based on the quantitative analysis, the two top-performing and two bottom-performing EMS agencies were selected for qualitative inquiry. A trained qualitative researcher conducted a total of 28 interviews with EMS agency leadership and staff at four rural EMS agencies in North Carolina. At each of the four sites, seven interviews were conducted. Each interview participant was practicing during the time of the quantitative data collection (2016–2019) used for ranking. Key informants included the director, training officer, medical director or assistant medical director, and four paramedics. Key informants were blinded to their agency’s STEMI performance-ranking and the reason for their agency’s inclusion in the study. Interviews were structured to explore participants’ clinical approach to patients with chest pain, the agency’s organizational culture, structure, and quality improvement (QI) activities regarding STEMI care, as well as recommendations for improving STEMI performance. The interview guide was pilot tested with four EMS clinicians from a local urban EMS agency and iteratively revised prior to data collection. Please see supplemental documents 1–4 for the complete interview guides. The interviews, which were conducted via videoconferencing software or telephone, ranged from 45–68 minutes, with an average length of 58 minutes. Interviews were digitally recorded and transcribed verbatim by a professional transcription service. A member of the research team reviewed all transcripts against the audio recordings for quality.

### Qualitative Analysis

We used Atlas.ti v9.0 (Lumivero, LLC, Denver, CO) to manage data.^43^ Thematic analysis was performed using an inductive approach.^44^ Two members of the research team systematically reviewed interview transcripts and identified relevant concepts and codes. Once an initial codebook was established, two researchers (AES and JPS) independently coded each transcript; they met regularly to discuss and resolve coding discrepancies and to revise the codebook, as needed. Data were then summarized within and across codes, compared across agencies to identify commonalities and differences between top- and bottom-performing agencies, and organized into themes that reflect the characteristics of high- and low-performing agencies’ clinical practices, organization culture, and QI activities. Themes were reviewed and validated by an expert panel composed of an EMS compliance officer, an emergency physician board-certified in EMS, and a hospital cardiovascular EMS liaison.

## RESULTS

We identified 365 patients for analysis whose demographics are available in Supplemental Table 1. Across the eight EMS agencies, the median number of STEMI cases per agency during the study period was 16 (IQR 11–40). The top two EMS agencies cared for 186 and 8 patients, respectively, with STEMI, while the bottom two EMS agencies cared for 11 and 13 patients with STEMI. The two high-performing EMS agencies had a median first medical contact-to-PCI time of 79 minutes (IQR 65–91) minutes vs 98 minutes (IQR 82–120) among the two low-performing agencies, *P*<.001. Compared to the bottom two agencies, the top two agencies were significantly faster in the following time intervals: ECG transmission to catheterization lab activation time (12.5 minutes [IQR 7–21] vs 21.0 [IQR 15–44], *P*=.002), on-scene time (14.0 minutes [IQR 11–16] vs 18 minutes [IQR 13–21)], *P*=.010], transport time (24.0 minutes [IQR 19–27] vs 38.0 minutes [IQR 28–42], *P*<.001), and total EMS time (37.0 minutes [IQR 32–42] vs 52.0 minutes [IQR 46–59], *P*<.001. Interestingly, door-to-PCI times were similar across agencies. See Table 1 for all time interval comparisons of top- and bottom-performing agencies. Supplemental Table 1 describes county- and agency-level factors for each EMS agency. Supplemental Table 2 describes patient- and incident-level factors stratified by whether PCI was achieved within the time goal. Fifty-five codes were identified from the key informant interviews and narrowed to five key themes. Key themes and representative quotes are shown in Table 2.

### Theme 1: Top agencies recognize the need for early STEMI activation

Paramedics and administrators in both high- and low-performing agencies commonly cited the importance of early activation of the cath lab following STEMI recognition. Top agencies emphasized the importance of paramedics expressing confidence when activating a STEMI and accepting that the physicians at the PCI center may disagree. Bottom agencies felt that emergency physicians were reluctant to notify the cath lab based on a paramedic’s interpretation of an ECG. Several paramedics in the low-performing agencies described lacking confidence in activating the cath lab themselves and a desire to defer to the judgment of a physician.

*We encourage them to err on the side of—be aggressive in STEMI alerts ... That’s one instance where time really matters with downstream outcomes. – A*dministrator, top agency

### Theme 2: Top agencies value quality improvement and collaboration with local hospitals

Both high- and low-performing agencies identified hospital involvement as valuable in QI, such as providing outcome data, sending pictures of patient’s vessel occlusions, and STEMI coordinators attending EMS peer-review meetings. Top agencies tended to consistently provide feedback to their paramedics who viewed feedback as a positive learning opportunity. In one of the low-performing agencies, paramedics stated they only received feedback if they asked for it. Others noted weeks- and months-long delays on receiving feedback. Top agencies tended to strive for consistent QI, whereas low-performing agencies tended to excuse missed metrics if an explanation was given in the patient care narrative. Top agencies recognized that good outcomes are a system responsibility and focus on systems issues, whereas the low-performing agencies focused on individual responsibility and clinician issues.

*Good medics really love to know what they found when they got to the cath lab. [Hospitals are] good to share that information. It keeps our medics involved and want to do good. –* Administrator, top agency

### Theme 3: Top agencies prioritize mission and chain of command over staff relationships and interpersonal bonds

Most participants from all agencies spoke positively about their agency culture. Top-performing agencies tended to describe an emphasis on high-quality patient care, staff wellness, following a chain of command, adherence to protocol, and service. Low-performing agencies had less emphasis on chain of command and protocols and seemed more focused on interpersonal relationships. For example, the low-performing agencies tended to reference a tightknit and family-like culture, pride in their agency, and open-door policies with administration.

*I think that our beliefs are patient oriented and service oriented—or at least that’s what I’m trying to instill in the organization. They’re told, when they’re hired, that the S in EMS stands for service, and that’s what we’re here to do and provide a service for the citizens*. – Administrator, top agency

### Theme 4: Top agencies have focused leadership and opportunities for career advancement

The high-performing agencies identified leadership that closely communicated with each other. Even when leaders were stretched thin, they prioritized crew needs. One top agency had a defined pathway for career advancement based on performance. The low-performing agencies identified difficulty with frequent leadership changes.

*…what [our county] has done is they’ve put in a career ladder to find opportunities for advancement within the agency to get rid of this idea, well it’s a good old boy network … it’s as objective as it can possibly be*. – Administrator, top agency

### Theme 5: Communication with the hospital and emergency department delays are a challenge for all agencies

Paramedics from both high- and low-performing agencies identified challenges communicating with hospitals. One EMS clinician from a top agency reported encountering incivility in encounters with ED staff and delays when attempting to consult with emergency physicians. Some paramedics preferred to bypass the ED and proceed directly to the cath lab. Paramedics from top agencies believed patients’ arrival to the cath lab were delayed by hospital staff not listening to them and performing unnecessary tasks, such as changing defibrillator pads. Paramedics from the low-performing agencies cited delays related to registration and frustration when patients were not taken directly to the cath lab. Technological difficulties, such as with transmitting ECGs, affected both the high- and low-performing agencies.

*What really usually creates a large problem is whatever charge nurse is on duty at that facility acts a little frustrated that, ‘You’re bothering me. What do you want?’ We have to go through that couple of minute delay…* – EMS Clinician, top agency

## DISCUSSION

This mixed-methods study identified potential drivers of performance differences for patients with STEMI observed between high- and low-performing rural EMS agencies. The top two and bottom two agencies differed significantly in first medical contact-to-PCI time after adjustment for distance from the PCI center. Most patients with a STEMI who were cared for the low-performing agencies did not meet the guideline-recommended first medical contact-to-PCI goal of <90 minutes, exposing them to increased risk of morbidity and mortality.^2^,^13^,^14^ Top-performing agencies shared a culture of early EMS activation of the cardiac cath lab, placed a high value on QI and training, prioritized mission and chain of command, and had stable leadership. Several key findings are detailed below.

First, we found differences in practice regarding STEMI recognition and cath lab activation. The median cath lab activation time among the top two agencies in this study was significantly faster than the bottom two agencies. Prehospital cath lab activation is an essential component of optimizing timely care for STEMI patients, and activation time is associated with door-to-PCI time.^34^,^46^–^48^ Top agency administrators and paramedics identified early recognition of STEMI and early cath lab activation as important. They tended to be aggressive in activation, accepting that false activations will occur and are acceptable. Conversely, the need for review of an ECG by a physician prior to cath lab activation was common among the low-performing agencies in this study. While the AHA Mission Lifeline PreAct algorithm recommends physician review of ECGs prior to cath lab activation, this practice may be detrimental.^49^ Lastly, paramedics from the low-performing agencies in our study discussed lack of confidence in diagnosing STEMI. Systems of STEMI care should continue to support prehospital cath lab activation without imposing additional barriers.^34^,^46^–^48^

Our second key finding relates to communication. First responders from all EMS agencies mentioned frustration with hospital communication and processes, with concerns ranging from delays in registering patients to incivility from nurses and physicians. Paramedics from both high- and low-performing agencies described technological barriers to communication, such as difficulty transmitting ECGs. Some paramedics strongly preferred proceeding directly to the cath lab rather than going through the ED. The safety and efficacy of the direct-to-cath lab strategy has been described repeatedly in the literature and is recommended by the AHA.^50^–^53^,^54^ Protocolization of care and improved communication may improve the intersection of EMS and in-hospital care.

Third, top agencies value designated and empowered QI officers who provide prompt performance feedback and training. As many as one in four US EMS agencies do not have dedicated QI personnel.^55^,^56^ Moreover, rural agencies are less likely to follow quality metrics than non-rural agencies.^56^ Top agencies in this study held a mature view of QI by recognizing that good outcomes are a responsibility of the system. Meanwhile, low-performing agencies focused on individual performance. Agencies should incorporate an evidenced-based, comprehensive continuous QI policy with sufficient resources to affect positive change. Key components of an effective QI program include clear aims, a non-punitive culture, focused education, and a teams-based approach.^55^

The impact of leadership was our fourth key finding. Strong leadership in healthcare organizations is associated with high quality care.^57^,^58^ The top agencies in our study described their leadership as focused, whereas the low-performing agencies described difficulty with leadership turnover. Nationally, turnover of EMS agency leaders is high, with a near 20% annual turnover rate nationally.^59^ It may be difficult for EMS agencies to adhere to a strong organizational culture of continuous improvement with frequent leadership turnover. Conversely, a mission-driven, outcome-oriented leadership culture has been associated with higher rates of job dissatisfaction among paramedics.^60^ Therefore, to improve patient care outcomes, leaders must be mindful of practices that may worsen job absenteeism and turnover.

Our prior article that reports the quantitative data used in this study describes a number of systems factors that contribute to delayed reperfusion.^23^ In descending order of strength of association, those factors associated with a lower odds of meeting first medical contact-to-PCI included female sex, cath lab activation outside normal business hours (ie, 5 pm −6 am), lack of exertional chest pain, longer distance transport, older age, and higher body mass index. The results of this study add key qualitative data that provide a framework of organizational structure, culture, and processes associated with better first medical contact-to-PCI performance among rural EMS agencies in North Carolina.

These data suggest that governmental and organizational leaders should support EMS agencies in developing focused, mission-oriented leaders and encourage collaboration between EMS agencies and hospitals. The EMS agency leaders should establish a clear chain of command, promote a mission-driven vision for their agency, and develop a collaborative and non-punitive QI and continuing education program. Paramedics should follow defined protocols for care of patients with chest pain and STEMI, including being comfortable activating the cardiac cath lab early in a patient’s care. Our data suggest that hospital-based healthcare professionals should collaborate with EMS agencies on QI and take action to improve interactions with paramedics that enhance collegiality and respect and build greater understanding of the limitations inherent to prehospital care. As process improvement is an iterative process, this study represents the first step in identifying opportunities to improve care. Future study will include hypothesis testing among a larger sample and subsequently working with stakeholders to disseminate results.^38^

## LIMITATIONS

This study ranked agencies using a database of patients from EMS systems that use a single electronic health record and have agreed to share their de-identified data for the purposes of research and benchmarking. These methods may have created participation bias as successful agencies may have been more likely to agree to participate in a data-reporting study than lower performing EMS agencies. Patients in this study were suspected of STEMI by their EMS clinician and not confirmed STEMI patients. Likewise, our analyses were limited to available data. Other confounding variables such as EMS staffing and experience and accuracy of documentation could not be assessed. Information in key-informant interviews may be susceptible to social desirability bias. However, an independent interviewer was used, and agencies were blinded to their rank.

The small number of employees for each agency completing an interview may limit generalization. Despite this, purposive sampling was used to include a diverse selection of employees. Included EMS agencies are in the southern US and may not be generalizable to STEMI patients in all EMS systems. Finally, the top-performing agency in our study cared for nearly half of the included patients while the lowest-performing agency cared for the fewest patients with STEMI per capita. Given that the association of hospital and procedural patient volume with improved outcomes has been extensively described,^61^^–64^ these agencies’ performance could be primarily driven by STEMI volume.

## CONCLUSION

Several key themes distinguished top-performing agencies, including understanding the need for early STEMI activation, recognizing the value of a strong quality improvement program, coordination with local hospitals, and mission-driven leadership. The EMS agencies may focus on a clear chain of command and promote a mission-driven vision for their agency supported by a collaborative QI program. Policymakers can support leadership development in EMS agencies. Paramedics should follow defined protocols for care and likely should be comfortable activating the cardiac catheterization lab early. Hospital-based healthcare professionals may work on improving interactions with paramedics when receiving patients with a suspected STEMI and should collaborate with EMS agencies on QI. Given the limitations of this article, future study is needed to evaluate these findings among a larger sample of rural EMS agencies.

## Supplementary Information















## Figures and Tables

**Table 1:** Time intervals among the two highest- and two lowest-performing emergency medical services (EMS) agencies for time from first medical contact to percutaneous coronary intervention among patients with ST-elevation myocardial infarction.

	Highest two ranked agencies (N=194) Median (IQR)	Lowest two ranked agencies (N=24) Median (IQR)	Difference in location^1^ (95% CI)	P-value^2^
FMC to PCI, minutes (PCI Time – At Patient)	78.5 (65.25–91)	97.5 (82.25–119.75)	−20.0 (−31.0, −10.0)	<.001
Dispatch Time, minutes (Dispatch – Call Received)	1 (0–2)	1 (1–2)	−1.0 (−1.0, 0.0)	.05
Response Time, minutes (On Scene – Dispatch)	9 (7–13)	11.5 (9.75–13.25)	−2.0 (−4.0, 0.0)	.04
Time to First ECG, minutes (12-Lead ECG – At Patient)	4 (2.75–5)	5 (2–9.5)	−0.0 (−2.0, 1.0)	.49
Activation Time, minutes (Cath lab activation – 12-lead ECG)	12.5 (7–21)	21 (15–44)	−10.0 (−17.0, −4.0)	< .01
Scene Time, minutes (Depart Scene – On Scene)	14 (11.25–16)	18 (12.75–21.25)	−3.0 (−6.0, −1.0)	.01
Transport Time, minutes (At Destination – Depart Scene)	24 (19–27)	38 (28–42)	−14.0 (−18.0, −10.0)	<.001
Total EMS Time, minutes (At Destination – On Scene)	37 (32.25–42)	52 (45.75–59)	−16.0 (−20.0, −12.0)	<.001
Door-to-balloon Time, minutes (PCI Time – At Destination)	41 (33–54)	37.5 (32–66.25)	−1.0 (−9.0, 6.0)	.85

1 Hodges-Lehmann estimator for the shift in location of the distribution of times for patients in the highest two ranked agencies compared to the lowest two ranked agencies,

2 Wilcoxon rank-sum test.

*IQR*, interquartile range; *CI*, confidence interval; *FMC*, first medical contact; *PCI*, percutaneous coronary intervention; *ECG*, electrocardiogram; *STEMI*, ST-segment elevation myocardial infarction.

**Table 2:** Themes and representative quotes from qualitative interviews of agencies leaders and paramedics.

Theme	Representative quotes
1. Top agencies recognize the need for early STEMI activation and are supportive of occasional false activation.	*We encourage them to err on the side of—be aggressive in STEMI alerts ... That’s one instance where time really matters with downstream outcomes. – A*dministrator, top agency *…to be honest with you, I don’t care what they [hospitals] see on the other end. I have enough feeling in my conscience to say, “This is a STEMI.” We’re going to run it as a STEMI. They can cancel it when we get there*. – Paramedic, top agency *This was an instance at [hospital], actually, where my 12-lead wouldn’t transmit. I called and talked to a doc, and I’m like, “Doc, I can’t get this 12-lead to transmit through, but this is what I’m lookin’—” and I explain to him a textbook inferior wall MI. From there, he said, “Well, hey, you’re my eyes and ears. You just identified one very well. I’m gonna go ahead and activate the cath lab,” and that went well*. – Paramedic, top agency *I can’t jump straight on the phone and start callin’. Sometimes, it may be 5, 10 minutes before I can get caught up just to make that phone call. I know sometimes that phone call’s requested immediately, but sometimes that can’t happen in the back of the truck in reality*. – Training officer, bottom agency *…this is a topic that paramedics feel just a little bit uncomfortable with ‘cause we’re being asked to ask for the cath lab to be activated and “is that really our decision” is what goes through our mind*. – Paramedic bottom agency *We preach to them that you don’t need to transmit the ECG but we all know it works better if you do. [Laughter] That’s just the way it is. We tell them that they [hospitals] should actively just based on your interpretation, but we all know they don’t. –* Administrator, bottom agency
2. Top agencies value quality improvement officers, training, and coordination with local hospitals.	*Good medics really love to know what they found when they got to the cath lab. [Hospitals are] good to share that information. It keeps our medics involved and want to do good. –* Administrator, top agency *Usually it’s like a shift meeting. We’ll talk about if it was extended scene times or—we’re always looking at ways to improve it. It’s not a punitive thing whenever we discuss somethin’. It’s more a learning opportunity, ‘Okay, this happened. How can we improve this? –* Paramedic, top agency *Communication is probably the top thing between EMS and the STEMI facilities because you’ve gotta have that connection, or it’s gonna fail…it started out bumpy. Anytime you implement somethin’ new, you’re gonna pick up some bumps. It took us a little while to get there, but I think now, especially in the last two or three years, we’ve had those representatives here. They were physically coming to our peer-review meetings and havin’ open, candid conversations about all the processes and havin’ the ability just to call ’em up and say, “Hey, what happened with this call, or what happened with this?” Either way, whether it’s us callin’ them, or them callin’ us. I think it’s improved*. – Administrator, top agency *We have a quality improvement officer…he reviews this type of stuff, and he don’t micromanage it in my opinion. ... If you’re on scene for a long time, all you have to do is justify it in your paperwork. If it’s justifiable, which it should be, then you’re fine to go. –*Paramedic, bottom agency *…that’s why I like that [hospital name] email because we can just forward that email, and honestly, they’ve already done the work. They’ve got EMS scene times on there. They’ve got the first 12-lead, the transmission time. It’s all there, so it makes it easy for us to be able to just share that with the employee and say, ‘Look, here’s your outcome on this call,’ and discuss those metrics as they happen, not every three months. –* Training officer, bottom agency *No one looks forward to being told, “Hey, you can do this a little better,” but they’re not as opposed or shamed about it because it’s an open way we do it. It’s the way we do business. They know that I’m not gonna jump up and down and scream and shout at ’em and take their pay for a day or suspend ’em. –* Administrator, bottom agency
3. Top agencies prioritize mission and chain of command over staff relationships and interpersonal bonds.	*I think that our beliefs are patient oriented and service oriented—or at least that’s what I’m trying to instill in the organization. They’re told, when they’re hired, that the S in EMS stands for service, and that’s what we’re here to do and provide a service for the citizens*. – Administrator, top agency *[X] County is a little more regimented in its culture, not necessarily in a negative way. The leadership … has a military background. There’s a lot more emphasis on chain of command, on following proper procedure, not oppressively so, but it’s the way they do things there.” –* Training officer, top agency *You’re told from the moment you’re hired in [X] County: We are a county service. We’re not privately owned, and we are the only advanced service available in [X] County, as far as there’s no hospital. We’ve got a smattering of doctor’s offices, and there are two…urgent care clinics. That’s it, so our director wants everyone to understand … there’s no one else you can call. There’s nowhere that you can take people within [X] Count y… these people rely very heavily on us*. – Paramedic, top agency *The culture, we still try to keep it as close-knit, extended, and family-type culture…we have open-door policies and things where if you need somebody to talk … just because we are the administration doesn’t mean you can’t come talk to us… –-* Administrator, bottom agency *We’ve had little instances pop up here and there, and we email back and forth—or, mostly, just up to them45—with no real feedback or response. At the end of the day, very few things end up getting accomplished that we ask for improvement from. –* Paramedic, bottom agency *Many of ’em are really close, so that’s important for our service to have that camaraderie among the service and especially among the shifts. That’s important for us, and that’s always been here at [county] since I’ve worked here is bein’ really tight. Everybody gets along really well. It’s not just a number. You don’t feel like you’re a number when you work here. We’re a small enough service that thankfully, everything’s personal here” …* – Paramedic, bottom agency
4. Top agencies have focused leadership and opportunities for career advancement.	*…what [county] has done is they’ve put in a career ladder to find opportunities for advancement within the agency to get rid of this idea, well it’s a good old boy network … it’s as objective as it can possibly be*. – Administrator, top agency *… when the COVID money started disbursing out to the county …our director and operations manager went immediately and bought the PAPR units. … We had ’em quick. Working in another county, I realized quickly how nice it was to have bosses that were doing everything they could to keep me safe …* –- Paramedic, top agency *There’s definitely a lot of room to be able to move up in the county. I think a lot of people try and stay for the most part ’cause it is a good county to work with*. – Paramedic, top agency *My four years as training officer, I’ve worked under three different directors. –* Administrator, bottom agency *We’re a small service, so we have a lotta roles. Between me and the training officer, we have a lot on our plate*. – Administrator, bottom agency *It’s physically taxing. It’s mentally taxing. Sometimes, the body just can’t hold up with it, or there’s some people and they just have had a lot of crummy calls that just weigh on ’em. They decide this isn’t really something I want to do anymore. I want to go do something that’s a little less taxing, and so they might find a different job*. – Paramedic, bottom agency
5. Communication with the hospital and emergency department delays are a challenge for all agencies.	*What really usually creates a large problem is whatever charge nurse is on duty at that facility acts a little frustrated that, ‘You’re bothering me. What do you want?’ We have to go through that couple of minute delay…* –- Paramedic, top agency …*it depends on the cardiologist. Some of ‘em really care about what we have to say. Some of them hear what we’ve done and then they just wanna talk with the patient* – Paramedic, top agency *I think the best thing to do is figure out a way that we could go directly to a cardiologist, review—you don’t have to be on the phone with that doctor, but it would be nice to be able to cut out the charge nurses or the ED because they are very busy already*. – Paramedic, top agency *First thing we do is have to sit there and wait for registration to get the patient registered. Even if we provide the patient information over the phone to the charge nurse, a lot of the times, they don’t have ‘em registered when we get there, even though they have access to all that information* – Paramedic, bottom agency *…we really appreciate whenever we call and ask to go to the cath lab that they take us directly there* – Paramedic, bottom agency *It can be frustrating sometimes. It’s an active STEMI, but maybe—I don’t know all the situations, but maybe the cath lab’s already busy, things like that, and we take him to a room. That can sometimes be a little bit frustrating ’cause they’re like—now, they’re just sitting down in the ED when we know we need to go to the cath lab, so that can be a little bit frustrating. I don’t know all the details behind why there’s that delay*. – Paramedic, bottom agency

*EMS*, emergency medical services; *ECG*, electrocardiogram; *STEMI*, ST-segment elevation myocardial infarction; *ED*, emergency department; *PAPR*, powered air-purifying respirator.
