# Supplementary material for: Mixed-Methods Investigation of Rural Emergency Medical Services ST-Elevation Myocardial Infarction Time to Percutaneous Coronary Intervention: High- vs Low-Performing Agencies
Source: West J Emerg Med. 2025 Jul 18;26(4):924–35. doi: 10.5811/westjem.43536 (PMC12342413; doi:10.5811/westjem.43536)
Supplement: Supplementary file 2 [file wjem-26-924-s002.docx]

EMS Medical Director Interview Guide

**MEDICAL DIRECTOR Interview Guide**

**Rural disparities in prehospital STEMI**

Thank you for agreeing to talk to us about the process of caring for patients that call 911 that are found to have a STEMI. Our goal is to understand the parts of rural EMS agencies’ organizational culture that influence the first medical contact to PCI time. We want to understand both the obstacles and the facilitators to achieving STEMI time goals. We will be talking to EMS Directors, EMS Training officers, Field Paramedics, EMT Crew Partners, and EMS Medical Directors at four of our local rural EMS agencies. Your feedback will help direct our strategy for developing an intervention to improve the prehospital care of rural patients with STEMI first here in NC and then nationally.

Did you receive the information form we sent to you?

Do you have any general questions for me before the interview begins?

Do you consent to participate and agree to have it recorded?

<START Audio & Video RECORDING>

I’ve just started the recorder. This is [interviewer name] and I’m speaking with XXX at XXX (site). Can you confirm if you consent to this audio-recorded interview?

**First, I would like to learn more about you and your role.**

1. Briefly describe your title and role as it relates to <site>.
2. How long have you been at <site>?
3. How long have you been in this role at <site>?
4. What is your background/ how did you become an EMS medical director?

**Now I’d like to ask some questions designed to explore your agency’s approach to prehospital chest pain care.**

1. Please describe <site’s> approach to patients that call 911 for a chief complaint of chest pain?
   1. How does the dispatcher decide which resources are sent to these calls?
   2. What priority do these calls carry for you? Do some chest pain calls carry a higher or lower priority for you?
2. Please describe how you want your paramedics (& EMTs) to approach patients with chest pain once they are on scene.
3. What are the first things that you want them to do? (NOTE: Want to see if they bring up time at all – do they realize that all chest pain patients need to be screened with EKG – do NOT bring up 10 minute EKG time goal for all patients with chest pain at this point)
   - - [IF RESPONDENT BRINGS UP 10 MIN EKG TIME GOAL] How do you ensure that your field providers meet the EKG time goal?
     - [ASK ALL] Do you review 12 Leads that are transmitted to receiving hospitals? Why or why not?
4. How should your field provider’s process change when a chest pain patient becomes a STEMI patient?
5. (NOTE: Want to see if they bring up time goals: BUT please don’t ask specifically here)
6. What is your agency’s scene time goal? (less than 10 or 15 minutes?)
7. How important is it that field providers meet the scene time goal?
   - - Ie is it more important to get IV first or get off scene?
8. How do you encourage or ensure that field providers achieve your agency’s scene time goal?
9. How frequently do providers have trouble communicating with the PCI centers while caring for a STEMI patient?
10. What are the sources of the communication difficulty? (Trouble activating – trouble connecting or too long to activate? Trouble transmitting ECG? )
11. In what scenarios, if any, would your agency consider going to a non-PCI center?

**Now, I’m going to ask a few questions about your field providers’ training and level of comfort with providing STEMI care.**

1. Tell me about your agency’s training in regard to chest pain and STEMI?
2. Is the training taught specific to your agency or at the more generic state level?
3. What is the focus of the training?
4. How often do trainings occur?
   - - Who leads them?
5. How comfortable or uncomfortable do you think your providers are with performing STEMI care?

**Next, I’m going to ask a few questions about the culture of your EMS agency**. For this study, we’re defining culture as the shared beliefs and values that are established by leaders, and then communicated and reinforced to employees, and that shape employee perceptions, behaviors, and understanding.

1. How would you describe the culture of your EMS agency?
   1. What is your relationship or interaction with the field providers?
   2. What is your relationship or interaction with the EMS Director?
2. How would you describe your agency’s communication among leadership?
3. How would you describe your agency’s communication from leadership to providers?
4. What changes, if any, would you like to see to improve the culture of the agency for field providers?

**My last few questions are STEMI performance goals and quality improvement activities.**

1. How has your agency’s approach to STEMI patients changed over time?
   1. What changes have you implemented, or tried to implement, around STEMI care?
   2. Have you tried to implement anything that wasn’t successful?
2. How clear are the STEMI performance goals for your agency?
3. How much of a priority is STEMI care for your agency?
4. How does that affect the resources you do or don’t get?
5. How well does your agency perform in achieving PCI time goals?
6. What makes it hard for your agency to achieve PCI time goals?
7. What helps your agency achieve PCI time goals?
8. (ANSWER: FMC-PCI time goal <90 min; time goal is increased to 120 minutes if transport time is >45min; transmit the EKG within 10 minutes of the ECG being obtained; activate within 10-15 minutes)
9. What do you think would increase your agency’s percentage of achieving FMC to PCI time goals?
10. Does your agency perform Chest pain QI?
11. If so, what does it entail?
12. Who is in charge of it?
13. Does your agency perform STEMI QI?
14. If so, what does it entail?

**We have about 5 minutes left and I want to make sure I give you an opportunity to let us know something about rural STEMI care that is important to you or your agency that I may have missed.** Is there anything that you would like to share that I haven’t asked about?

This is the end of the interview!

Thank you for your time. We will be sending your gift card to the address you provided us in the interview scheduling email in the next 3 business days.
