# Supplementary material for: Mixed-Methods Investigation of Rural Emergency Medical Services ST-Elevation Myocardial Infarction Time to Percutaneous Coronary Intervention: High- vs Low-Performing Agencies
Source: West J Emerg Med. 2025 Jul 18;26(4):924–35. doi: 10.5811/westjem.43536 (PMC12342413; doi:10.5811/westjem.43536)
Supplement: Supplementary file 3 [file wjem-26-924-s003.docx]

EMS Training Officer Interview Guide

**TRAINING OFFICER Interview Guide**

**Rural disparities in prehospital STEMI**

Thank you for agreeing to talk to us about the process of caring for patients that call 911 that are found to have a STEMI. Our goal is to understand the parts of rural EMS agencies’ organizational culture that influence the first medical contact to PCI time. We want to understand both the obstacles and the facilitators to achieving STEMI time goals. We will be talking to EMS Directors, EMS Training officers, Field Paramedics, EMT Crew Partners, and EMS Medical Directors at four of our local rural EMS agencies. Your feedback will help direct our strategy for developing an intervention to improve the prehospital care of rural patients with STEMI first here in NC and then nationally.

Did you receive the information form we sent to you?

Do you have any general questions for me before the interview begins?

Do you consent to participate and agree to have it recorded?

<START Audio & Video RECORDING>

I’ve just started the recorder. This is [interviewer name] and I’m speaking with XXX at XXX (site). Can you confirm if you consent to this audio-recorded interview?

**First, I would like to learn more about you and your role.**

1. Briefly describe your title and role as it relates to <site>.
2. How long have you been at <site>?
3. How long have you been in this role at <site>?
4. What state level medical certification do have? How long have you been a <certification>?

**Now I’d like to ask some questions designed to explore your agency’s approach to prehospital chest pain.**

1. How do you train your agency’s paramedics (& EMTs) to approach patients with chest pain once they are on scene.
   1. What are the first things that you want them to do? (NOTE: Want to see if they bring up time at all – do they realize that all chest pain patients need to be screened with EKG - do NOT bring up 10 minute EKG time goal for chest pain patient at this point)
      - [IF RESPONDENT BRINGS UP 10 MINU EKG TIME GOAL] How do you ensure that your agency’s field providers meet the EKG time goal?
      - Do you review 12 Leads that are transmitted to receiving hospitals? Why or why not?
2. How should your agency’s provider’s process change when a chest pain patient becomes a STEMI patient?
3. (NOTE: Want to see if they bring up time goals: BUT please don’t ask specifically here)
4. In what scenarios, if any, wuld your agency consider going to a non-PCI center?

**Now, I’m going to ask a few questions about the agency’s training, STEMI performance goals, and quality improvement activities.**

1. Tell me about the training your agency’s filed providers receive on chest pain and STEMI?
2. Is the training taught specific to your agency or at the more generic state level?
3. What is the focus of the training?
4. How often do trainings occur?
   - - Who leads them?
5. What is your agency’s scene time goal? (less than 10 or 15 minutes?
6. How important is it that field providers meet the scene time goal?
   - - Ie is it more important to get IV first or get off scene?
7. How do you encourage or ensure that field providers achieve the agency’s scene time goal?
8. How clear are the STEMI performance goals for your agency?
9. How well does your agency perform in achieving PCI time goals?
10. What makes it hard for your agency to achieve PCI time goals?
11. What helps your agency achieve PCI time goals?
12. (ANSWER: FMC-PCI time goal <90 min; time goal is increased to 120 minutes if transport time is >45min; transmit the EKG within 10 minutes of the ECG being obtained; activate within 10-15 minutes)
13. What do you think would increase your agency’s percentage of achieving FMC to PCI time goals?
14. How has your agency’s approach to STEMI patients changed over time?
    1. What changes has your agency implemented, or tried to implement, around STEMI care?
    2. Have you tried to implement anything that wasn’t successful?
15. Does your agency perform Chest pain QI?
16. If so, what does it entail?
17. Who is in charge of it?
18. Does your agency perform STEMI QI?
19. If so, what does it entail?

**My next few questions are about the culture of your EMS agency.** For this study, we’re defining culture as the shared beliefs and values that are established by leaders, and then communicated and reinforced to employees, and that shape employee perceptions, behaviors, and understanding.

1. How would you describe the culture of your EMS agency?
   1. How is information communicated within the agency? Feedback?
   2. What is your relationship or interaction with the field providers? EMS Director? Medical Director?
   3. How much staff turnover does your agency have? Why/ why not?
2. What changes, if any, do you think would improve the culture of the agency?
3. How would you describe your agency’s communication among leadership?
4. How would you describe your agency’s communication from leadership to providers?

**We have about 5 minutes left and I want to make sure I give you an opportunity to let us know something about rural STEMI care that is important to you or your agency that I may have missed.** Is there anything that you would like to share that I have not asked you about? (If not, it’s ok, I will keep asking the questions I have prepared.)

[If there is time:]

1. How much of a priority is STEMI care for your agency?
2. How does that affect the resources you do or don’t get?
3. How comfortable or uncomfortable do you think your providers are with performing STEMI care?
4. How frequently do providers have trouble communicating with the PCI centers while caring for a STEMI patient?
5. What are the sources of the communication difficulty? (Trouble activating – trouble connecting or too long to activate? Trouble transmitting ECG?)

This is the end of the interview! Thank you for your time. We will be sending your gift card to the address you provided us in the interview scheduling email in the next 3 business days.
