# Supplementary material for: Mixed-Methods Investigation of Rural Emergency Medical Services ST-Elevation Myocardial Infarction Time to Percutaneous Coronary Intervention: High- vs Low-Performing Agencies
Source: West J Emerg Med. 2025 Jul 18;26(4):924–35. doi: 10.5811/westjem.43536 (PMC12342413; doi:10.5811/westjem.43536)
Supplement: Supplementary file 4 [file wjem-26-924-s004.docx]

Paramedic Interview Guide

**Paramedic Interview Guide**

**Rural disparities in prehospital STEMI**

Thank you for agreeing to talk to us about the process of caring for patients that call 911 that are found to have a STEMI. Our goal is to understand the parts of rural EMS agencies’ organizational culture that influence the first medical contact to PCI time. We want to understand both the obstacles and the facilitators to achieving STEMI time goals. We will be talking to EMS Directors, EMS Training officers, Field Paramedics, EMT Crew Partners, and EMS Medical Directors at four of our local rural EMS agencies. Your feedback will help direct our strategy for developing an intervention to improve the prehospital care of rural patients with STEMI first here in NC and then nationally.

Did you receive the information we sent to you?

Do you have any general questions for me before the interview begins?

Do you consent to participate and agree to have it recorded?

<START Audio & Video RECORDING>

I’ve just started the recorder. This is [interviewer name] and I’m speaking with XXX at XXX (site). Can you confirm if you consent to this audio-recorded interview?

**First, I would like to learn more about you and your role.**

1. Briefly describe your title and role as it relates to <site>.
2. How long have you been at <site>?
3. How long have you been in this role at <site>?
4. What state level medical certification do have? How long have you been a <certification>?
5. Do you work at another EMS agency? If so, which one(s)? What is your role? How often do you work there?

**Now I’d like to ask some questions about your approach to prehospital chest pain care.**

1. Please describe your approach to patients with a chief complaint of chest pain.
2. What is the protocol for assessing a chest pain patient? What is the sequence of things that you do? (NOTE: Want to see if they bring up time at all – do they realize that all chest pain patients need to be screened with EKG – do NOT bring up 10 minute time goal for chest pain patients at this point)
   - - [IF RESPONDENT BRINGS UP 10 MIN TIME GOAL] How important is the 10 minute EKG time goal?
       1. How difficult is it to achieve the 10 minute EKG time goal?
       2. Why?
     - [ASK ALL] What things do you find yourself doing before getting 12 Lead EKG?
     - Do you get the 12 lead EKG or ask someone else to get it?
     - How often is the first 12 lead EKG of adequate quality?
3. How does your process change when a chest pain patient becomes a STEMI patient?

(NOTE: Want to see if they bring up time goals: BUT please don’t ask specifically here)

1. Why does your process change?
   - - (CORRECT ANSWER: Assembly of cath lab team and lab prep is the rate limiting step especially after business hours ---- Better outcomes for quicker PCI)

**Next, I am going to ask about communication with the PCI and patient transport.**

1. Tell me about a time when you had trouble with communication with the PCI center while you were caring for a STEMI patient.
2. How frequently do you have trouble communicating with the PCI center?
3. What are the sources of the communication difficulty? (Trouble activating cath lab (communicating with the PCI center so they can call in the cath team so the patient can get timely PCI) Trouble connecting? Trouble transmitting ECG?)
   1. Probe for specific PCI centers (Moses Cone, Novant, High Point, Baptist)
4. What is your process of establishing the route to the PCI center?
5. Who is responsible for establishing the route?
6. What factors into decision making?
7. Tell me about a time that you or your driver took the wrong route.
8. How often is your transport delayed while en route?
9. What are the reasons for those delays? (ie stop because patient becomes unstable or ambulance equipment failure)
10. In what scenarios, if any, would you consider going to non-PCI center?

**Now I’m going to ask you a few questions about the culture of your EMS agency.** For this study, we’re defining culture as the shared beliefs and values that are established by leaders, and then communicated and reinforced to employees, and that shape how employees perceive the work and the mission of the agency, as well as employee behaviors and actions.

1. How would you describe the culture of your EMS agency?
   1. How is the culture of your own shift similar to or different from the overall agency?
   2. How is information communicated within your agency? Feedback?
   3. What is your interaction or relationship with the Medical Director, EMS Director?
2. What changes, if any, would you like to see to improve the culture of your EMS agency?
   1. What changes, if any, have taken place at the agency since you started?
   2. How much confidence do you feel that you have to be able to speak up about a challenge, or to affect change?
3. How does communication with the other responders play into your on scene activities and decisions?
4. How does teamwork play into your on scene activities and decisions?

**Now, I’m going to ask a few questions about your agency’s STEMI performance goals, and training and quality improvement activities.**

1. What is your agency’s scene time goal? (less than 10 or 15 minutes?)
2. How important is it that you meet the scene time goal?
   - - Probe: is it more important to get IV first or get off scene?
3. What do you do to make sure you achieve your agency’s scene time goal?
4. What are some reasons why you may stay on scene longer than your agency’s scene time goal?
5. How does communication with the patient play into your on scene activities and decisions?
6. How well do you perform in achieving PCI time goals?
7. What makes it hard for you to achieve PCI time goals?
8. What helps you achieve PCI time goals?
9. What part of the 90 minutes time goal has the most opportunity to improve?
10. How, if at all, do you receive information about your PCI time? Do you follow up on your times?
11. What do you think would increase your percentage of achieving first medical contact to PCI time goals?
    1. What role, if any, do you think county government would have in increasing your percentage of achieving FMC to PCI time goals?
    2. What role, if any, do you think the EMS administration/training staff have in increasing FMC to PCI time goals?
    3. What, if any additional resources do you need to increase your percentage of achieving FMC to PCI time goals? Crew? Tools?
12. Does your agency perform STEMI QI? If so, what does it entail?
13. Tell me about the training that you’ve received with regard to chest pain and STEMI.
14. Was the training specific to your agency, or was state level training?
15. What is the focus of the training?
16. How often do trainings occur? (When was the last time you received training?)
17. How comfortable or uncomfortable are you with performing STEMI care?

**We have about 5 minutes left and I want to make sure I give you an opportunity to let me know anything about rural STEMI care that is important to you or your agency that I may have missed.** Is there anything that you would like share that I have not asked about? (If not, it’s ok, I will keep asking the questions I have prepared).

[If there is time:]

1. Please describe how your agency approaches patients that call 911 for a chief complaint of chest pain?
   1. What priority do these calls carry for you? Do some chest pain calls carry a higher or lower priority for you?

24. What equipment do you bring to the chest pain patient?

- - - Describe a time when you didn’t bring equipment to the patient but instead brought the patient to your ambulance where the equipment was.

25. On average, how long does it take you to get from where you park your ambulance on scene to patient contact?

- - - What are some of the things that make this faster or slower?

26. How often do you have rescue/first responders on chest pain calls?

1. How does this impact your efficiency?

27. How does having a second paramedic impact your efficiency?

1. How often do you have two paramedics?

This is the end of the interview! Thank you for your time. We will be sending your gift card to the address you provided us in the interview scheduling email in the next 3 business days.
