# Supplementary material for: Mixed-Methods Investigation of Rural Emergency Medical Services ST-Elevation Myocardial Infarction Time to Percutaneous Coronary Intervention: High- vs Low-Performing Agencies
Source: West J Emerg Med. 2025 Jul 18;26(4):924–35. doi: 10.5811/westjem.43536 (PMC12342413; doi:10.5811/westjem.43536)
Supplement: Supplementary file 5 [file wjem-26-924-s005.docx]

**Supplemental Table 1**: Rural STEMI patient baseline characteristics.

|  | **TotalN = 365 (%)** | **Patients with PCI within goal n = 221** | **Patients with PCI beyond goal n = 144** | **p-value*** |
| --- | --- | --- | --- | --- |
| **Age – years, mean ± SD** | 62.5 ± 12.7 | 60.9 ± 12.6 | 64.9 ± 12.5 | 0.04 |
| **Sex (Female)** | 110 (30.1) | 48 (21.7) | 62 (43.1) | <0.001 |
| **Race/Ethnicity** |  |  |  | 0.90* |
| **White** | 340 (93.2) | 206 (93.2) | 134 (93.1) |  |
| **Black** | 18 (4.9) | 10 (4.5) | 8 (5.6) |  |
| **Other** | 7 (1.9) | 5 (2.3) | 2 (1.4) |  |
| **BMI kg/m2, mean ± SD** | 29.4 ± 5.9 | 29.2 ± 5.3 | 29.9 ± 6.8 | 0.02 |
| **Current Smoking** | 168 (52.0) | 106 (52.7) | 62 (50.8) | 0.61 |
| **Hypertension** | 250 (68.7) | 139 (62.9) | 111 (77.6) | 0.047 |
| **Hypercholesterolemia** | 223 (61.1) | 126 (57.0) | 97 (67.4) | 0.13 |
| **Diabetes** | 106 (29.0) | 55 (24.9) | 51 (35.4) | <0.001 |
| **Prior CAD** | 106 (29.1) | 56 (25.3) | 50 (35.0) | 0.002 |
| **Cancer (current or history of)** | 41 (11.3) | 18 (8.1) | 23 (16.1) | 0.04 |
| **Provoked onset with activity** | 77 (21.1) | 58 (26.2) | 19 (13.2) | <0.001 |
| **Pain Characteristics** |  |  |  | 0.03* |
| **None** | 16 (4.8) | 5 (2.5) | 11 (8.5) |  |
| **Pressure/Burning/Tightness** | 66 (19.8) | 41 (20.1) | 25 (19.4) |  |
| **Sharp/Stabbing** | 67 (20.1) | 45 (22.1) | 22 (17.1) |  |
| **Other** | 184 (55.3) | 98 (44.3) | 57 (39.6) |  |
| **First SBP Category** |  |  |  | 0.88 |
| **<90 mmHg** | 26 (7.4) | 16 (7.4) | 10 (7.4) |  |
| **≥90 mmHg** | 325 (92.6) | 200 (92.6) | 125 (92.6) |  |
| **First Pulse Rate, bpm** |  |  |  | 0.003 |
| **<60** | 55 (15.2) | 33 (15.0) | 22 (15.4) |  |
| **60-100** | 250 (68.9) | 157 (71.4) | 93 (65.0) |  |
| **>100** | 58 (16.0) | 30 (13.6) | 28 (19.6) |  |
| **First SpO2** |  |  |  | 0.56 |
| **<90%** | 9 (2.7) | 5 (2.5) | 4 (3.1) |  |
| **≥90%** | 319 (97.3) | 192 (97.5) | 127 (96.9) |  |
| **7a-5p or 5:01p-6:59a by ECG time (initial time point of activation time)** |  |  |  | <0.001 |
| **7:00 AM − 5:00 PM** | 197 (54.4) | 139 (62.9) | 58 (41.3) |  |
| **5:01 PM − 6:59 AM** | 165 (45.6) | 82 (37.1) | 83 (58.9) |  |
| **Loaded Mileage, mean ± SD** | 26.5 ± 11.0 | 24.7 ± 10.2 | 29.4 ± 11.7 | 0.02 |
