# Supplementary material for: Mixed-Methods Investigation of Rural Emergency Medical Services ST-Elevation Myocardial Infarction Time to Percutaneous Coronary Intervention: High- vs Low-Performing Agencies
Source: West J Emerg Med. 2025 Jul 18;26(4):924–35. doi: 10.5811/westjem.43536 (PMC12342413; doi:10.5811/westjem.43536)
Supplement: Supplementary file 6 [file wjem-26-924-s006.docx]

**Supplemental Table 2.** Factors for the eight enrolled EMS agencies with rank as top or bottom agency.

| Agency Ranking | Population  2018 | Annual patient transports | Annual patient transports per 100 citizens | Number of full-time paramedics | Number of staffed ambulances | STEMI Cases Included |
| --- | --- | --- | --- | --- | --- | --- |
| Top Agency | 143,176 | 10,600 | 7 | 40 | 9 | 186 |
| Top Agency | 37,456 | 3500 | 9 | 35 | 5 | 8 |
| Bottom Agency | 45,459 | 6000 | 13 | 34 | 5 | 11 |
| Bottom Agency | 71,957 | 17,000 | 24 | 73 | 7 | 13 |
| - | 22,691 | 2,600 | 11 | 12 | 3 | 10 |
| - | 42,504 | 4,600 | 11 | 54 | 5 | 18 |
| - | 90,680 | 9700 | 11 | 58 | 8 | 99 |
| - | 68,481 | 8400 | 12 | 52 | 5 | 20 |

STEMI = ST-segment Elevation Myocardial Infarction
