# Supplementary material for: Mixed-Methods Investigation of Rural Emergency Medical Services ST-Elevation Myocardial Infarction Time to Percutaneous Coronary Intervention: High- vs Low-Performing Agencies
Source: West J Emerg Med. 2025 Jul 18;26(4):924–35. doi: 10.5811/westjem.43536 (PMC12342413; doi:10.5811/westjem.43536)
Supplement: Supplementary file 7 [file wjem-26-924-s007.docx]

**
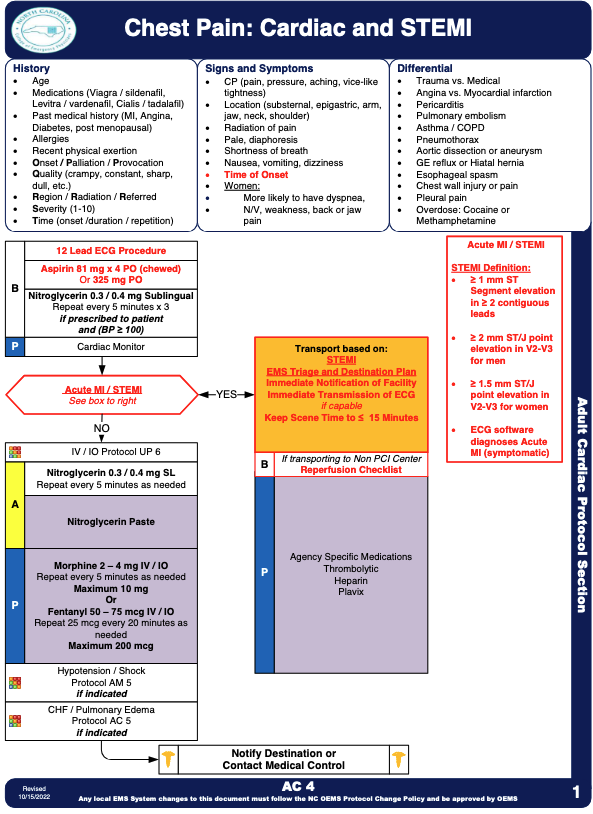
**

**Supplemental Figure 1.** North Carolina Office of Emergency Medical Services chest pain, cardiac and ST-elevation myocardial infarction protocol.

*MI,* myocardial infarction; *CP,* chest pain; *N/V,* nausea/ vomiting; *COPD,* chronic obstructive pulmonary disease; *GE,* gastroesophageal; *ECG,* electrocardiogram; *PO,* by mouth; *BP,* blood pressure; *STEMI,* ST-segment elevation myocardial infarction; *IV,* intravenous, *IO,* intraosseous; *AM,* adult medical; *AC,* adult cardiac; *CHF,* congestive heart failure; PCI, percutaneous coronary intervention; *UP,* universal protocol; *SL,* sublingual.
